# How machine learning is shaping cognitive neuroimaging

**DOI:** 10.1186/2047-217X-3-28

**Published:** 2014-11-17

**Authors:** Gael Varoquaux, Bertrand Thirion

**Affiliations:** 1 Parietal, INRIA, NeuroSpin, bat 145 CEA Saclay, 91191 Gif sur Yvette, France

**Keywords:** Machine learning, Neuroimaging, Cognition, fMRI, Decoding, Encoding

## Abstract

Functional brain images are rich and noisy data that can capture indirect signatures of neural activity underlying cognition in a given experimental setting. Can data mining leverage them to build models of cognition? Only if it is applied to well-posed questions, crafted to reveal cognitive mechanisms. Here we review how predictive models have been used on neuroimaging data to ask new questions, i.e., to uncover new aspects of cognitive organization. We also give a statistical learning perspective on these progresses and on the remaining gaping holes.

## Introduction

Functional neuroimaging has opened the door to quantitative yet non invasive experiments on brain function. These experiments contribute to bridging the gap between cognitive sciences and neuroscience: the former analyse thought and mind while the latter probes the nervous system at various spatial and temporal scales. To study high-level aspects of human cognition, the two modalities of choice are functional Magnetic Resonance Imaging (fMRI) and electro-and magneto-encephalography (EEG/MEG), both can be used to observe brain activity with good spatial resolution for fMRI and temporal temporal resolution for EEG/MEG. The concurrent progress of scanners and experimental paradigms has made it possible to accumulate very rich imaging data that quantify specific correlates of brain function in an uncountable variety of cognitive tasks and processes. In parallel, the advent of machine learning has brought huge progress to data processing of large datasets. But these techniques are geared towards well-posed predictive tasks. The key question is then; how can they be leveraged to push forward understanding of the brain, beyond merely predicting a numerical signal?

This paper presents a subjective view on the work that has been done combining machine learning with functional neuroimaging to advance the understanding of brain function. It dwells mostly on modeling considerations: how and what do the predictive models teach us about the brain? But it also touches upon machine learning and statistical issues. This review focuses on fMRI in humans, that represents most of the accumulated functional neuroimaging data; however, most of the concepts carry to other imaging modalities. FMRI provides images of the brain at the mm scale, however it is only sensitive to the metabolic counterpart of neural activity and suffers from a poor temporal resolution. The first two sections of this paper discuss supervised learning, used first to model brain activity from the stimuli, then to predict the task performed from the evoked activity. The last section reviews the use of unsupervised learning to extract relevant structures in functional images: the interaction structure that underlies brain function, or their natural spatial organization.

## Encoding: richer models of evoked activity

The keystone to the use of fMRI in cognitive neuroscience is the standard mass-univariate analysis framework. It consists of modeling the brain response evoked via an experimental paradigm as the linear combination of different experimental conditions [1,2]. A statistical test is performed at each voxel to delineate regions recruited differently by the various conditions. The art of fMRI experiment design and analysis then consists in crafting the succession of conditions so that, when properly contrasted, they reveal the neural support corresponding to the cognitive function of interest. With regards to brain function, this statistical analysis answers naturally a “where” question, but to a lesser extent a “how” question. Indeed the tests for differences between experimental conditions are statistically well-posed, but not very expressive to refine cognitive models.

In contrast, the study of *neural coding*, lead historically via intra-cellular recordings of neural activity, has opened the door to breaking down many cognitive functions into atomic steps implemented by ensembles of neurons. The seminal work of Hubel and Wiesel [3] showed that neurons in the primary visual cortex have *receptive fields* tuned to a variety of image features, from simple cells sensitive to local orientation in an image, to more complex cells capturing in addition, motion and length of local image features. Progress on uncovering the link between stimuli and neural response revealed neurons tuned to richer and higher-level descriptions of the stimulus, such as receptive fields specific to complex shapes [4], but also a richer description of neural responses, in particular coding distributed across a population of neurons [5].

Beyond individual neurons, at the spatial scales probed in fMRI^a^, and high-level cognition arises from functional integration of multiple specialized brain regions [7].

The stepping stones of this line of work are to find the right features of the stimuli and neuronal population that can be matched closely. How well the former explains the latter gives a natural figure of merit of these models, in a setting known as *encoding*[8]. Given models that explain neural responses at the spatial scales captured by fMRI [9,10] rather than at the neural level, encoding research can be lead with fMRI data, which benefits from full-brain coverage. Technically, designing an encoding model is not different from specifying the design matrix in a standard fMRI analysis and can be seen as *model-based fMRI*[10]. However relinquishing the methodology of contrasts for more diverse, albeit indirect, statistical tests opens the door to richer modeling. In particular, it is possible to tackle more complex stimuli, such as natural stimuli [11], very high-level and diverse descriptions of the stimuli [12], or a cognitive model of the observed behavior [10].

This increase in model complexity is the driving force behind the use of machine learning in encoding. First it entails fitting many parameters on limited data, and thus conventional in-sample statistical testing is thorny. For this reason, goodness of fit of the encoding model is best assessed via its cross-validated ability to predict brain signals [13]. Similarly, the predictive engine that links stimuli features to brain signal is best chosen amongst machine learning tools, that balance modeling flexibility and regularization, such as a naive Bayes predictor [12], sparse [13] or ridge [14] regression. Finally, the computational models that derive encoding features from the stimuli often draw from the domain-specific feature extraction techniques developed in applied machine learning research. These provide simple quantitative proxies for the cognitive features of interest. For instance, to map semantic concepts [12] and [14] used natural language processing techniques: word co-occurrence or an ontology on words. The ties between brain science and machine learning are strikingly close in the study of vision: computer vision, *i.e.,* the use of computers and machine learning to analyze and interpret images, has built upon, but also fostered our understanding of the brain visual system. David Marr’s seminal work [15] formalized the idea of hierarchical levels of representation that tie together the receptive fields observed in visual cortex, but is also reflected in modern state-of-the-art computer vision architecture based on convolutional networks [16]. Very recently, Yamins *et al.*[17] have shown a striking correspondence between 296 neural recordings in the infero-temporal cortex of the monkey and intermediate layers of computer-vision convolutional networks. This work is a quintessential example of machine learning in encoding models: a predictive engine performs the same task as the brain system under study; machine learning is used to fit its parameters on a set of stimuli and the final architecture matches neural data.

Transferring such results to fMRI would open doors to studying the full complete brain of healthy human subjects rather than 296 neurons in implanted monkeys. However, it poses significant challenges. Indeed, fMRI is an indirect and noisy measurement of brain activity, that captures the average effect of many spikes and does not resolve cortical columns, let alone individual neurons. The concept of *population receptive field*[18] is sometimes used to refer to the aggregate properties of neurons in one voxel. Thus, encoding models need to be adapted to the resulting structured noise and signal convolutions. Model evaluation and selection is in itself often a major roadblock.

## Decoding: towards principled reverse inference

In the study of neural recordings, decoding models reconstruct stimuli or behavior from the neural code [5,19]. More generally, the decoding approach can be seen as solving the inverse problem to the encoding model, even when applied on fMRI signals that do not capture individual neural firing [20,21].

Since a decoding model often predicts quantities that are directly observable, it can provide a very useful tool to validate an encoding model. Indeed, decoding performance is an omnibus test of goodness of fit: it tests the overall significance of the model, but does not test which variables have a significant contribution to the model. As an omnibus test, decoding is used with explicit sophisticated encodings [8,11-14], but also with simple fMRI analysis to perform an omnibus test at the region level [22] or on a wide family of regions as in searchlight analysis [23]. Interestingly, an early fMRI study [9] on neural representation hypothesized that “objects are represented by a relatively widely distributed activity of functional modules”, but considered this statement to be insufficiently quantitative to allow a statistical test. Nowadays this study would probably be formulated in an encoding/decoding framework [8], using a multivariate predictor to provide evidence for the author’s hypothesis, as in [22]. It is often considered that multi-voxel analysis, as used in decoding, provides an increase in sensitivity compared to standard mass-univariate analysis [24]; however, we stress that it does not correspond to an increase in statistical power, but rather to a different test performed: decoding performs a global (omnibus) test of the model, while voxel-level tests are useful to delineate regions, but are subject to corrections for multiple comparisons.

As noted in [25], decoding analysis provides a good framework to interpret overlapping activation patterns. Brain maps in encoding and decoding settings carry actually a different meaning. An inference with an encoding model, or in the fMRI standard analysis framework, is a statement on whether or not the signal in a brain region is well explained by the model that we have of the task: we can conclude that the task implies this brain activation, and we say that the region is *recruited* by the task. A decoding analysis tells us that if we observe a certain brain activity, we can deduce properties of the task or the stimulus. Such a conclusion is the converse implication of the encoding settings, sometimes dubbed *reverse inference*[26]. Reverse inference, *i.e.,* drawing conclusions on behavior and mental processes from the brain activations, answers natural questions in cognitive neuroimaging, *e.g.,*: what is the function of neural sub-system? But reverse inferences drawn from maps, estimated using encoding models, are a logical fallacy [26]. On the other hand, decoding models provide a path to principled reverse inferences [27]. However, it is important to keep in mind that, in general, a decoding experiment does not tell us anything about tasks and cognitive processes that it did not probe. For example, an experiment studying brain regions discriminating images of faces from images of houses [22] does not inform us on how these regions are related to recognizing letters.

The appealing idea of inferring brain processes from brain activation only carries meaning if the decoding model has captured a large variety of brain processes. Beyond interpretation of brain images, the basic neuroscience questions at stakes here are that of functional specificity. For instance, while many brain regions are more activated under physical pain, a decoding analysis including many different aspects of pain showed that a network comprising parts of the thalamus, the insulae, and the somatosensory cortex was specific of physical pain [28]. At the spatial scale probed by fMRI, the multiplicity of regions needed to come to precise conclusions on the cognitive function recruited is consistent with the modern view that high-level cognitive processes arise from distributed networks. This calls for multivariate decoding engines.

Going beyond a specific cognitive domain, such as vision or pain, and studying functional specialization in a broad sense require probing more functions than can be addressed in one experimental paradigm. For this reason, investigators have turned to accumulating data across experiments. Using 8 studies, covering 22 different cognitive concepts, Poldrack *et al.*[29] were able to predict the concepts involved from activation images in unseen subjects. The use of a variety of studies, with different experimental protocols, can overcome the idiosyncrasies of each protocol that are not relevant to cognitive concepts of interest; for instance, to study high-level decision mechanisms independently of the modality used to present stimuli –visual or auditory. However, in [29], the train set contained images from the same protocols as the test set; thus, the hypothesis that the decoder was actually detecting protocols rather than cognitive concepts cannot be ruled out. To generalize to unseen protocols, the challenge is to describe them in terms that are common enough to be shared across many protocols, but also sufficiently rich to capture their cognitive content. Schwartz *et al.*[30] used an ontology of experimental paradigms and multi-label classification: labeling 83 different conditions, from 19 studies, with a set of different terms from the ontology. The resulting predicting engine can not only describe the content of an unseen experiment from the corresponding brain activation, but also give brain maps associated with each term in a reverse inference. Covering more cognitive concepts requires accumulating many brain images. Sharing data across institutions is a practical means to this end, for instance relying on the OpenfMRI project [31] that hosts to this day 24 different fMRI studies. Another interesting alley is to collect from the literature the coordinates, in standard brain space, of observed activation foci, as in the Neurosynth project [32].

Although decoding gives a principled methodological framework for reverse inference, there are some tough statistical challenges. Indeed, the discriminant brain maps extracted may be the most relevant information captured by the model from a neuroscience perspective. However, decoders solve a high-dimensional multivariate statistical estimation problem that is very ill-posed [33] given the typical small sample size. Many different brain maps will give rise to similar predictive performance. Worst yet, minimizing a prediction risk does not lead to any control on the brain maps. For instance, if two neighboring voxels carry the same information but one is less noisy than the other, a decoder might favor selecting only that one. For related reasons, sparse models can only capture a subset of relevant voxels [34]. Injecting priors –or regularization– in the estimation makes it well-posed and shapes the brain maps extracted. Capturing large-scale brain systems calls for spatial regularization such as sparsity and spatial smoothness [35] or total-variation (TV) for piecewise smooth maps [36]. In particular TV- *ℓ*_1_ regularization, combining sparsity and total-variation, selects well the predictive regions [37]. Unlike widespread belief, multivariate tools used commonly, such as support vector machines or searchlight, seem to do a worse job at selecting predictive regions than univariate tools [37].

**Figure 1:**
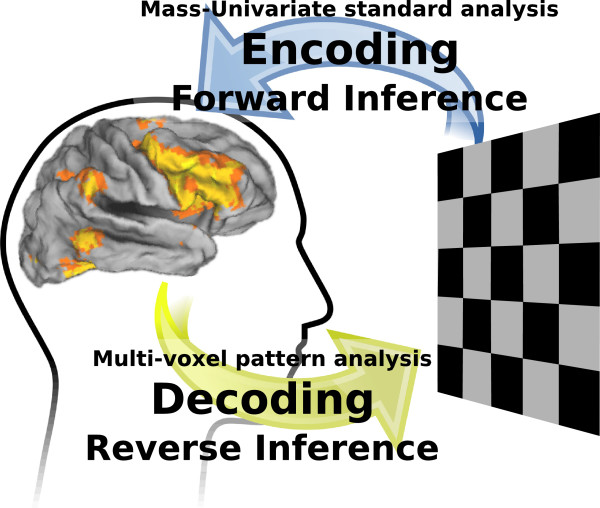
Schematics of the distinction between encoding and decoding in brain imaging.

Encoding and decoding models explore the two directions linking brain activation to stimuli and cognitive processes [8] (see Figure 1). Both of these methodologies do not form credible models of how the brain creates cognition. They are rather experimental devices to test hypotheses and retrieve brain maps, where the critical modeling work goes in the formal description of the cognitive concepts associated with the brain signals under study. This description is most often a non-trivial transformation of the stimuli, non-linear [17] or calling for concept ontologies [14,29,30]. Following the concepts of neural coding and Marr’s vision that good representations give rise to powerful computational processing [15], encoding and decoding models are often understood as revealing a *representational space*, distributed representations in the cortex that reflect fundamental concepts [9,38]. However, the combination of the lack of temporal dynamics in fMRI and the linear models that we rely upon naturally create such an understanding of the data in terms of *representations*, while for some functions studied, the actual neural implementation may be closer to *processes*[39] dynamically sustained information, as in theories of conscious processing [40]. In this light, the use of linear models for decoding may be criticized as too simple to capture non-linear interactions. However, from the neuroscience point-of-view they lead to probing well-posed questions [8] and from the statistical learning point of view, they can be relatively well-behaved even in very high dimensional settings with the typical small sample sizes faced by fMRI [34].

## Finding hidden structure: parcellations and connectomes

In machine learning applications, it is often easier to accumulate unlabeled data than labeled data. This is also the case in neuroimaging, as controlling the cognitive state of the subject is very challenging and calls for careful experimental design and analysis. Data collection speed is limited by the timescale of psychological experiments. On the opposite, accumulating so-called *resting-state* acquisitions, in which the mental state of the subject is not controlled, is much more tractable [41], and is applicable to diminished populations [42]. The interest of resting-state data for cognitive neuroimaging is not immediate, as it does not carry explicit cognitive information; however, they reflect *on-going* activity, which is an important part of brain function. Indeed, on-going activity shares a common substrate with activity explicitly evoked by controlled cognition, priming cognition but also shaped by task [43]. Unsupervised learning on resting-state scans holds the promise of extracting intrinsic brain structures [41].

### Capturing brain interactions

The brain is a heavily interacting system. Mapping its connections in the form of a *connectome*[44] can help to understand the flow of information in the brain. Fluctuations in brain activity, for example, observed at rest, reveal functional interactions and thus can be used to estimate a *functional connectome*[45]. From a statistical learning perspective, the estimation of a connectome can be formalized as extracting the conditional independence structure from observed correlations [46]. The challenge here is that of the paucity of data, and can be tackled with graph estimators that have good small-sample structure recovery properties, such as sparse covariance models [47,48].

### Learning functional units

Interest in resting-state data arose originally from the observation that voxel-based signals observed at rest could be used to segment spatial structures known from task studies [49]. Subsequently, researchers realized that these could exhibit some additional functional structures [50]. What spatial distributed brain networks are modulated during rest? This question can be formulated as that of blind source separation, and independent component analysis (ICA) provides a good algorithm to recover these networks [51,52]. Datasets of increasing size and quality lead to extracting more networks, that break up in a set of smaller regions, paving the brain in a parcellation [53]. Breaking down the brain into homogeneous units is a long quest in neuroscience that can be traced back to Brodmann areas. Such parcellations have been historically driven by anatomical features. Resting-state fMRI provides valuable data to learn a functional parcellation, as it gives a spatially-resolved window into intrinsic brain function. Indeed, functionally-homogeneous regions can be extracted by clustering voxels with similar fMRI time-series [54,55]. The unmixing model underlying ICA can be adapted to extracting regions by formulating it in the more general framework of dictionary learning [56] and adding sparsity-inducing penalty that also favor clustered spatial components, thus yielding region segmentations [57]. While identifying intrinsic functional brain modules is crucial from a basic neuroscience point of view, brain parcellation can also provide useful data reduction even if they don’t capture true functional units [21,34]. These different purposes give rise to different methodological trade-offs [58]. Beyond resting-state data, applying similar methods to databases of evoked activity exploring a large variety of cognitive concepts can have the additional benefit of appending cognitive labels to the spatial units extracted [59,60].

However, care must be exercised when applying the brain-parcellation techniques. By construction, such methods will return a parcellation, even if there is little to no structure in the data. They do not build upon well-posed statistical hypothesis testing. The methods can often be unstable, with a small modification of the input data leading to large changes in the results. This unstability can be explained by, on one hand the lack of explicit noise model, and on the other hand the fact that unsupervised learning is an intrinsically hard problem from the statistical standpoint. Validation of the functional units is very challenging beyond a simple confirmation bias that boils down to checking for known structures, the variability of which is unknown and uncontrolled. Some researchers have explored quantifying variability of the patterns [55,57,58,61] or controlling how well they explain the data [57,58] but these are weak proxys of the neuroscientific questions on brain organization.

## Practical considerations: methods and implementations matter

The focus of this review is not on methodological details, but on general concepts and approaches that further our understanding of brain function. However, it is important to stress that many of the roadblocks to the use of machine-learning-related techniques in cognitive neuroimaging lie in the methods. From a theoretical point of view, the statistical control is seldom warranted by the models used [34,37]. On the empirical side of things, best practices are not established. The high-dimensionality of the statistical models and the plurality of methods considered mean that, at the level of the literature, machine-learning techniques probably give rise to more variability, although they do come with more expressiveness and power.

A final critical aspect, all too often overlooked, is that of software. The standard GUI-based fMRI data processing environments, such as SPM, FSL [62] or AFNI [63], do not implement most of the modeling approaches described in this review. FSL and AFNI do provide some methods tailored to fMRI uses (respectively ICA [52] and basic decoding [64]). There is progress on dedicated tools such as PyMVPA [65], but these require the practitioners to learn new skills, in particular some understanding of machine learning and basic programming. The challenges of a good environment for machine-learning on fMRI data is that it should be simple enough to be within reach of the practitioner, yet leverage a powerful machine-learning toolkit, such as the popular *scikit-learn* package in Python [66], and offer flexibility to assemble new models, encoding, decoding, or unsupervised [67].

## Conclusions

The goals of cognitive neurosciences are to link cognition with its neural basis. FMRI gives a noisy and incomplete window on neural mechanisms. Nevertheless, to map effects at a large scale, it is priceless, as it can be applied massively on healthy human subjects, and thus enables the systematic study of high-level cognition. Machine learning tools are instrumental in making the most of this data, as they do not require a precise mechanistic understanding of the signal, but rather to frame a prediction problem that captures some relevant aspects of brain function, as in encoding or decoding. However, for progress in neuroscience, black-box prediction engines do not suffice as the key to understanding brain function lies in the properties of the signal used for prediction. For these reasons, the statistics aspects in statistical learning cannot be neglected: different methods give rise to different results and the figure of merit does not simply boil down to predictive power.

## Endnote

^a^ It is unlikely that standard fMRI acquisitions, even after analysis with powerful multivariate methods, capture information at the level of the cortical column [6].

## Abbreviations

fMRI: Functional magnetic resonnance imaging; EEG: Electro encephaloGraphy; MEG: Magneto encephaloGraphy; TV: Total-variation; ICA: Independent component analysis; GUI: Graphical User Interface.

## Competing interests

The authors declare that they have no competing interests.

## Authors’ contributions

GV and BT carried out the research and drafted the manuscript.
